# A role for Phospholipase D in *Drosophila *embryonic cellularization

**DOI:** 10.1186/1471-213X-6-60

**Published:** 2006-12-07

**Authors:** Mary LaLonde, Hilde Janssens, Suyong Yun, Juan Crosby, Olga Redina, Virginie Olive, Yelena M Altshuller, Seok-Yong Choi, Guangwei Du, J Peter Gergen, Michael A Frohman

**Affiliations:** 1 Department of Pharmacology and Center for Developmental Genetics, Stony Brook University, Stony Brook, NY 11794-5140, USA; 2 Department of Biochemistry and Center for Developmental Genetics, Stony Brook University, Stony Brook, NY 11794-5140, USA; 3 Institute of Cytology & Genetics, Siberian Branch of the Russian Academy of Sciences, Novosibirsk, Russia

## Abstract

**Background:**

Cellularization of the *Drosophila *embryo is an unusually synchronous form of cytokinesis in which polarized membrane extension proceeds in part through incorporation of new membrane via fusion of apically-translocated Golgi-derived vesicles.

**Results:**

We describe here involvement of the signaling enzyme Phospholipase D (Pld) in regulation of this developmental step. Functional analysis using gene targeting revealed that cellularization is hindered by the loss of Pld, resulting frequently in early embryonic developmental arrest. Mechanistically, chronic Pld deficiency causes abnormal Golgi structure and secretory vesicle trafficking.

**Conclusion:**

Our results suggest that Pld functions to promote trafficking of Golgi-derived fusion-competent vesicles during cellularization.

## Background

Embryogenesis in *Drosophila melanogaster *commences with 13 nuclear divisions in the absence of cytokinesis, generating a syncytium of ~6,000 nuclei located immediately beneath the plasma membrane. Cellularization then ensues, resulting in the synchronous formation of lateral and then basal membranes around each nucleus through rearrangement of the actin cytoskeleton and extensive formation of de novo membrane. It is estimated that a 25-fold increase in plasma membrane is necessary to complete the process. The de novo membrane comes from the resorption of microvilli on the outer surface of the blastoderm [1,2] and the incorporation of ER- and/or Golgi-derived secretory vesicles [3-7].

Genetic screens have identified numerous proteins that regulate cytoskeletal reorganization during cellularization, several proteins of unknown function including SLAM [7] that are required at the leading edge of the extending lateral membrane (the furrow canal), and a plasma-membrane associated component of the exocytic membrane trafficking machinery, the SNARE protein Syntaxin1 (Syx1A, reviewed in Mazumdar and Mazumdar, 2002). Both Syx1A and SLAM enable the fusion of incoming membrane vesicles into the expanding membranes. In the absence of SLAM, membrane vesicles accumulate in the apical cytoplasm. However, the accumulating vesicles contain Rab11, a marker for recycling endosomes, suggesting that the incoming Golgi-derived secretory vesicles either first fuse into the apical plasma membrane, and then endocytosis and traffic through the recycling endosomal compartment prior to fusing into the expanding lateral membranes, or traffic directly to the lateral membranes via the Rab11 endosomal pathway [8].

Little is known about the nature of the incoming Golgi-derived secretory vesicles or how their apical trafficking is regulated, aside from the findings that Lava lamp (Lva), a microfilament/microtubule-associated protein that potentially couples the Golgi to the microtubule network [6], and the regulated small GTPase Rho [9,10] which is known for its role in cytoskeletal reorganization and membrane vesicle trafficking, are required for the progression of cellularization. Disruption or brefeldin A-mediated inhibiton of exchange factors for the small GTPase ARF1 also inhibits cellularization [6,11].

A protein that facilitates Golgi-derived vesicle production, trafficking, and fusion into target membranes in yeast and mammalian cells is the signaling enzyme Phospholipase D (PLD), which hydrolyzes the membrane phospholipid phosphatidylcholine to yield the lipid second messenger phosphatidic acid [reviewed in [12]]. PA is a pleiotropic lipid that functions in membrane vesicle trafficking by promoting membrane budding from the Golgi complex [13,14], by facilitating exocytic trafficking [15,16], and by promoting vesicle fusion into target membranes [17-19]. In addition, through regulation of phosphatidylinositol 4,5-bisphosphate (PI4,5P_2_) production, PLD/PA regulates actin cytoskeleton reorganization [20]. Isolation of PLDs from yeast and mammals led to the identification of an evolutionarily conserved family of genes [21-23]. Two mammalian PLD genes with approximately 50% identity exist, but only a single gene is present in yeast. Mammalian PLD activity is regulated by signal transducing pathways in a complex manner involving Protein Kinase C (PKC), Rho, and ARF, and in turn activates its own effector pathways [reviewed in [12]].

Lacking null mutants in animals, current knowledge concerning cellular roles for PLD in higher eukaryotes had largely come from overexpression or pharmacological inhibitor experiments in tissue culture systems. We have recently described the first such genetic model, using *Drosophila *that lack a functional PLD gene, and a role for PLD in phototransduction, a process that involves both signaling and vesicle trafficking [24]. Membrane trafficking as a means to expand or build specialized membranes is a common event in all animals, including during cytokinesis and neurite extension. We describe here a role for PLD in the cellularization pathway that generates and delivers Golgi-derived membrane vesicles to the embryonic cortex.

## Results

### A single phospholipase D (Pld) exists in Drosophila

**Figure 1:**
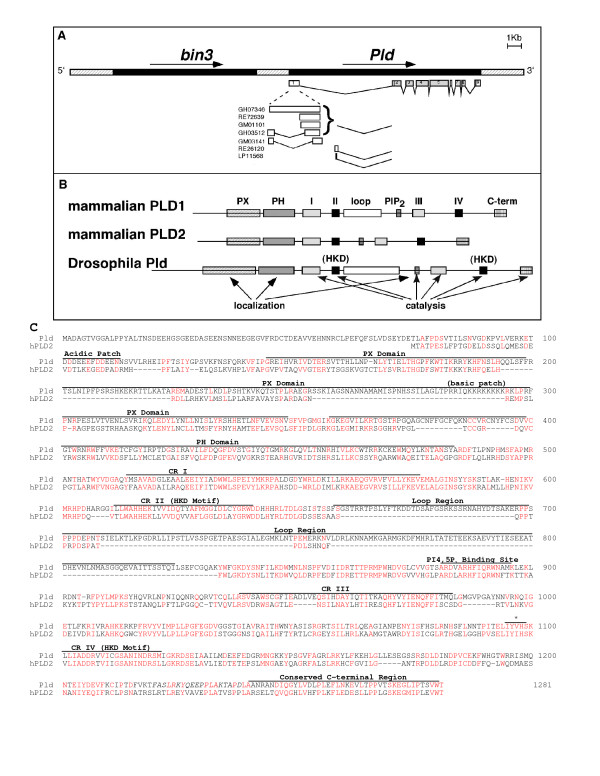
**Pld gene and protein structure**. A. The *Pld *genomic locus is shown (42A15), as well as the closely located *bicoid-interacting protein 3 *(*bin3*) region (black). The *Pld *transcript is represented as boxes with exon numbers inside. The coding region is indicated in gray. ESTs representative of different 5' UTR splice isoforms found in BDGP are shown (GH: adult head; RE: embryos 0–22 h; GM: ovaries; LP: larvae and early pupae). B. The Pld domain organization is shown in comparison with mammalian PLD1 and 2. C. Identical and highly similar amino acids in human PLD2 are highlighted in red. (*) indicates the position of the mutant amino acid in the Pld-H1095N allele.

Our initial report on the existence of a PLD gene family [22] described a *Drosophila *sequence-tagged site that encoded a protein fragment with similarity to human PLD1. A combination of genomic PCR, conventional screening of ovary cDNA libraries, and RACE-PCR was used to obtain *Drosophila Pld *cDNAs containing a complete open reading frame (*Pld*, accession number AF228314, AF228315, Fig. 1A). No other PLD-like sequences are present in the BDGP databases, and southern blot analysis using *Pld *as a probe under reduced stringency conditions did not identify additional hybridizing bands (data not shown), suggesting that there is only one PLD gene in *Drosophila*. The larva and pupa, adult head, and ovary *Pld *cDNA clones present in the BDGP database all code for the same amino acid sequence but vary in their 5'UTRs (Fig. 1A). 5'UTR usage did not correlate with specific developmental stages or tissue sources.

*Pld *encodes a 1278 amino acid protein that exhibits ~35% sequence similarity to human PLD2 (Fig. 1B, C), and slightly less to human PLD1. The protein contains the conserved regions characteristic of other members of the eukaryotic PLD family: a Phox homology (PX) domain followed by a Pleckstrin homology (PH) domain, conserved catalytic domains (CR I-IV), a loop sequence, a PI(4,5)P_2_-interacting motif, and a conserved carboxy terminus [reviewed in [12]]. The Pld amino-terminus is noteworthy for a non-conserved "acidic patch" (residues 101–112), whereas the PX domain, which in the mammalian isoforms regulates subcellular localization [16], contains an unusual "basic patch" (residues 283–298). PX domain functioning in Pld may be regulated uniquely by interactions between the acidic and basic patches. Although the central loop region in Pld is in the same position as the loop found in mammalian PLD1, it lacks similarity to it, suggesting that this region may serve as a hinge region between the two catalytic half-domains, as has been shown for the mammalian PLD enzymes [25], rather than to convey structural or functional information. Taken together, the presence of the characteristic PLD domains suggested that Pld should be a bona fide PLD homologue.

### Pld is activated by components of signaling pathways that are important in cellularization

Although yeast PLD is constitutively active (Rose et al., 1995), mammalian PLD is regulated by ARF and Rho GTPases. Accordingly, the report that PLD activity in *Drosophila *could be stimulated by GTPγS [26] suggested that *Drosophila *Pld might be regulated similarly. To investigate this, *Pld*, and a putatively inactive allele of *Pld *containing a point mutation (H1095N) in the second HKD domain that renders all other known PLD proteins inactive [25], were expressed in mammalian cells.

Mammalian PLD1 exhibits little activity in the absence of cellular stimulation [22]. PMA, a protein kinase C agonist that activates PLD1 through both direct and indirect pathways, potently increases PLD activity. In contrast, only a small increase in activity, reflecting activation of endogenous PLD, is observed when inactive PLD1 (K898R) is overexpressed and assayed [25]. Similarly, *Drosophila *Pld activity, but not Pld-H1095N activity, significantly increased following PMA stimulation [see supplemental figure in 24].

**Figure 2:**
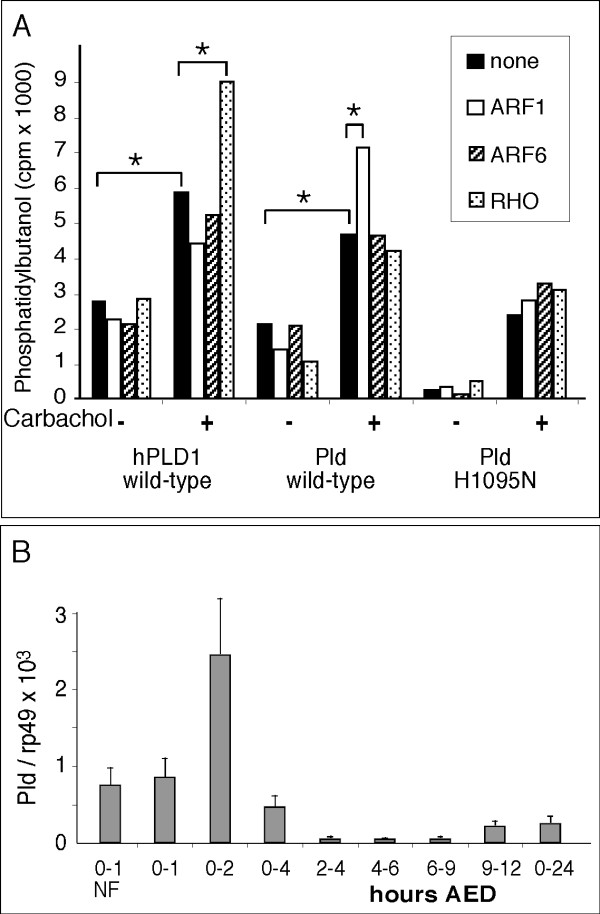
**Pld exhibits classical Phospholipase D activity regulated by signaling pathways and peaks in expression prior to cellularization**. A. HEK-293 cells stably expressing the m3 muscarinic acetylcholine receptor were co-transfected with *pCGN:Pld *and with ARF1, ARF6 or RhoA. After 24 hours, the cells were stimulated with carbachol, an activator of G-protein coupled muscarinic receptors, and assayed in vivo for PLD activity. The assay is based on the ability of PLD to use primary alcohols, such as butanol, in place of water, in the PC hydrolysis reaction. This leads to the production of phosphatidyl-alcohol (-butanol, PtdBut). Assaying PtdBut is preferable to attempting to quantitate PA, since PA is highly labile and turns over quickly, whereas PtdBut is relatively inert, accumulates over the course of the 30' assay, and thus provides an estimate of the total amount of PLD activity that took place during the assay period. See citation 41 for assay details. The data represent means of triplicate measurements. Standard deviations averaged 3–5%. The experiment is representative of three independent experiments. *, significant difference, p < 0.05. B. Temporal expression of endogenous Pld as analyzed by quantitative PCR. Embryonic stages are given in hours after egg deposition (AED). 0–1 NF, non-fertilized eggs between 0 and 1 hour AED. Cellularization occurs between 2 and 3 h AED during the 14^th ^mitotic cycle. *Pld *cDNA concentration is expressed as a ratio of *Pld *to *rp49*, a ribosomal RNA expressed at constant levels.

To determine whether ARF and Rho stimulate *Drosophila *Pld, we used a cell-based system in which increased levels of the GTPases exaggerate mammalian PLD responses to G-protein receptor-coupled signaling. HEK293 cells expressing the m3 muscarinic acetylcholine receptor [27] were co-transfected with *pCGN:Pld *and mammalian ARF1, ARF6, or RhoA expression plasmids (Fig. 2A). A small amount of activity was observed in cells overexpressing the inactive Pld-H1095N protein, which derived from the endogenous mammalian PLDs present in the HEK293 cells. Similar levels of activity are observed after transfection of inactive human PLD1 (hPLD1-K898R) or empty vectors (supplemental figure in citation 24 and data not shown). Wild-type *Drosophila *Pld was activated by receptor signaling through carbachol stimulation and this was enhanced by overexpression of ARF1, as has been observed for mammalian PLD2 (Du et al., 2000). In contrast, hPLD1 activity was enhanced more readily by overexpression of RhoA. Taken together, these findings demonstrate that Pld is a bona fide PLD and can be regulated by ARF GTPases, which mediate vesicle trafficking and actin cytoskeleton reorganization in many settings.

### Pld is maternally and zygotically expressed and peaks prior to cellularization

Many of the proteins required for cellularization are synthesized from maternal transcripts, although a few are transcribed zygotically and peak just prior to or at the onset of cellularization [28]. To determine the temporal sequence of *Pld *mRNA expression in early embryogenesis, we used quantitative RT-PCR (Fig. 2B). *Pld *is expressed maternally, since it was observed in the non-fertilized (NF) 0–1 hr after egg deposition (AED) collection. It is also expressed zygotically, based on the dramatic increase observed when zygotic transcription initiates, i.e. between the fertilized 0–1 hr and the 2 hr AED collection. *Pld *mRNA levels plummeted between 2 and 4 hours AED and increased again modestly around 9 hours AED. Thus, the temporal pattern of *Pld *expression is consistent with that of genes that have roles in cellularization.

### Pld localizes to small cytoplasmic vesicles during cellularization

In some mammalian cell types, PLD1 functions at sites of exocytosis on the plasma membrane to promote the fusion of secretory granules [18,19]. In others, though, PLD1 localizes to the Golgi and/or peri-nuclear vesicles and facilitates budding from the trans-Golgi to generate Golgi-derived secretory vesicles [e.g. [29]], inter-endosomal or ER-to-Golgi trafficking [30], or trafficking of secretory vesicles to the plasma membrane [15]. Determining where Pld localizes would therefore suggest potential roles for its function during cellularization.

**Figure 3:**
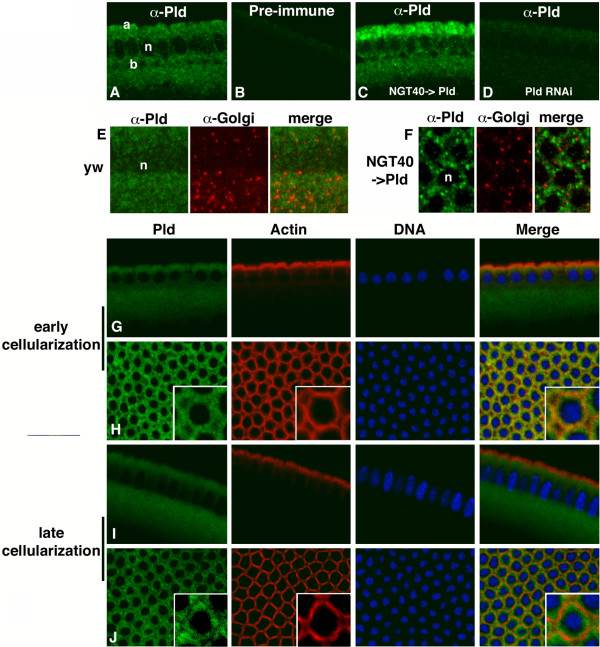
**Pld localizes to cytoplasmic vesicles during cellularization**. A. Pld localization in heat-fixed *y*^*1*^*w*^*67c23 *^control embryos using an affinity-purified anti-Pld antisera (sagittal image generated at or near the embryonic equator). a, apical; n, nuclei; b, basal. B. Affinity-purified pre-immune serum does not generate a visible signal. C. Pld localization in transgenic lines overexpressing Pld as a maternal transcript using *NGT-GAL4*. D. Pld localization in transgenic lines expressing Pld RNAi. E, F. Pld does not co-localize with p120-marked Golgi vesicles (red) in wild-type (sagittal section) or *Pld; NGT40+A *embryos (surface view). G-J. Cellularizing embryos immunostained with anti-Pld antisera (green), monoclonal anti-actin (red), and Propidium iodide (blue). Pld in early cellularizing embryos (G, H) co-localizes with actin at sites where adjacent cells are forming junctions (inset). Later in cellularization (I-J), Pld does not localize to sites at which actin tracks have been assembled (inset).

In cellularizing embryos, Pld localized to the cytoplasm and/or to cytosolic vesicles of uniform small size evenly distributed between apical and basal regions of the blastoderm (Fig. 3A–D). Pld was not observed to localize specifically to the plasma membrane, membrane furrows, or extending lateral membranes where membrane vesicle fusion takes place. Detection of Pld overexpressed by the NGT40 promoter (Fig. 3C) confirmed that the antisera recognizes Pld protein. When overexpressed, Pld exhibits a preferential localization for the apical region of the cell; the reason for this is not known but is suggestive with respect to the overexpression phenotype discussed below. Pld also did not co-localize with Rab11 as a marker for recycling endosomes (not shown) or the 120-kD Golgi integral membrane protein, p120 (Fig. 3E, F). During early cellularization, Pld partially co-localized with actin at the sites where cytoskeletal reorganization and membrane formation are taking place (Fig. 3G, H). This overlap ceased by the end of cellularization, as indicated by the clearing of Pld within the established actin tracks (Fig. 3I, J). Taken together, this pattern of localization would be consistent with a role for Pld in translocation of fusion-competent Golgi-derived vesicles to the embryonic cortex or regulation of the actin cytoskeleton, although roles in budding or fusion are not ruled out.

**Figure 4:**
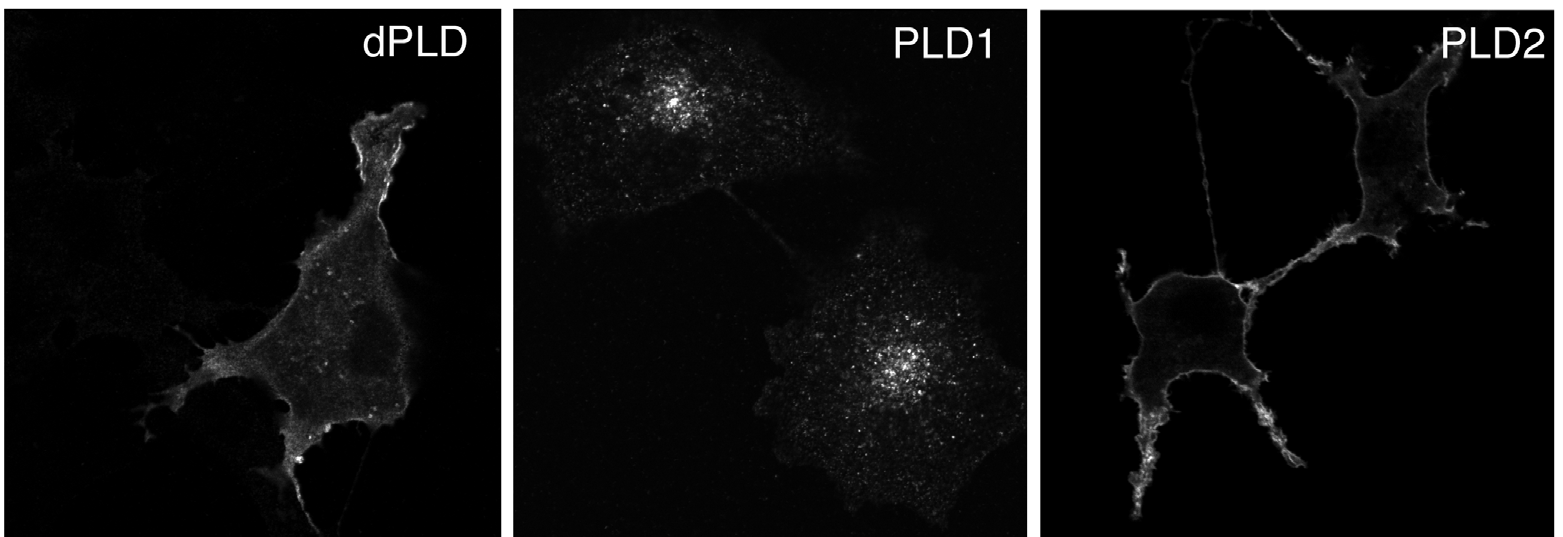
**Drosophila Pld recombinant protein localizes to cytoplasmic vesicles and the plasma membrane in COS7 cells**. COS7 cells were transiently transfected with either *pCGN:dPld*, *pCGN:mPLD1*, or *pCGN:mPLD2*. The cells were immunostained with a rat monoclonal anti-HA antibody (3F10). mPLD1 localized to peri-nuclear vesicles, while mPLD2 was predominantly localized to the plasma membrane. *Drosophila *Pld staining marked intracellular vesicles and the plasma membrane.

To compare the localization of *Drosophila *Pld to its mammalian homologs, *Drosophila *Pld was expressed in (mammalian) COS7 cells. Pld localized to cytoplasmic vesicles of varying size and to the plasma membrane (Fig. 4). The vesicular staining did not co-localize with the ER-marker calreticulin or the recycling endosome proteins Rab11 and the transferrin receptor (data not shown). The pattern of subcellular localization of dPld was intermediate between that of mammalian PLD1 and PLD2: PLD1 localized to peri-nuclear vesicles, whereas PLD2 localized to the plasma membrane. The unique aspects of the *Drosophila *PLD pattern of localization could ensue from the non-conserved regions within its amino-terminus and/or loop region.

### Pld overexpression during early embryogenesis reduces viability

The effect of increasing PLD activity during cellularization was examined using the *P{GAL4-nos.NGT} *transgenic binary expression system [31] to deliver wild-type or catalytically-inactive (H1095N) *Pld *at the start of embryogenesis. Immunostaining indicated that both isoforms were expressed at elevated levels, particularly in the apical region of the newly forming cells (Fig. 3C). Pld overexpression did not affect Golgi localization or morphology (Fig. 3E, F and data not shown). Embryonic lethality averaging 30.4% (in three experiments) was observed for all *P{UAS-Pld} *transgenic lines driven by the strong *GAL4 *driver *P{GAL4-nos.NGT}40; P{GAL4-nos.NGT}A*. Embryonic lethality was dependent on expression level since no lethality was observed with the weaker *P{GAL4-nos.NGT}11 *driver. In addition, lethality was not observed for the strong *GAL4*-driven overexpression of the catalytically-inactive *P{UAS-Pld-H1095N} *transgene, indicating that the phenotype ensues from increased PLD activity and thus PA production, rather than from a non-enzymatic consequence of PLD overexpression.

### Genetic ablation of Pld reduces viability in early embryogenesis

**Figure 5:**
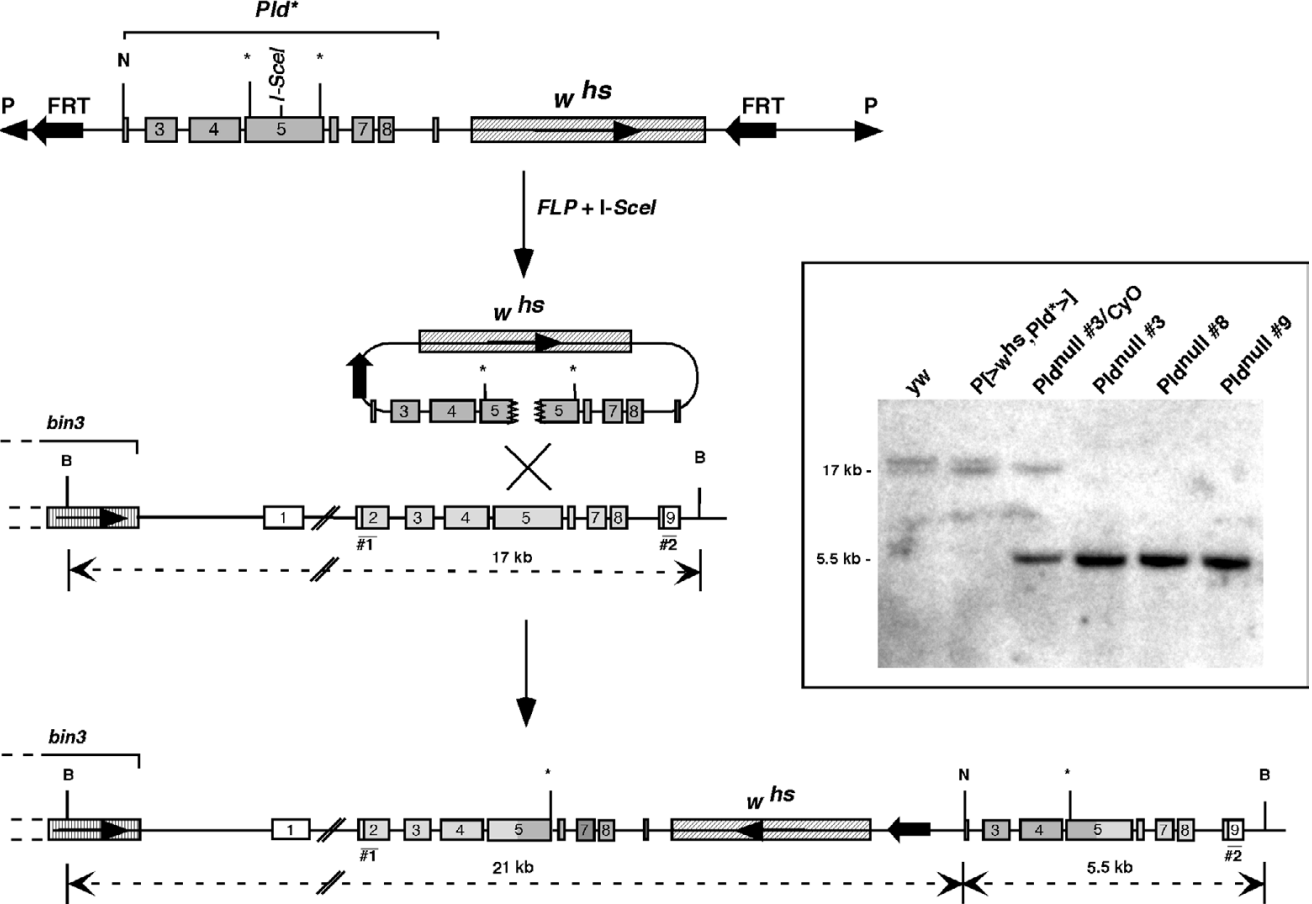
**Homologous recombination targeting strategy**. The donor construct *P{>w*^*hs*^,Pld*>} (top) lacks most of the second and ninth exons and contains two stop codons and a recognition site for I-*Sce I *in exon 5. The extrachromosomal targeting molecule is generated by FLP recombinase-mediated excision and I-*SceI *cleaving of the donor construct (middle). Alignment of the targeting DNA and the endogenous *Pld *locus by ends-in recombination results in two mutant copies of the *Pld *gene (bottom). Locations of relevant restriction sites and Southern blot hybridization probes used (#1 and #2) are indicated. The sizes of the different fragments detected by Southern blot analysis of the wild-type and mutated alleles are indicated beneath the genomic representations (dotted lines). B=*BamH I*; N=*Not I*. (Inset) Southern blot analysis of the *Pld *targeting event. Genomic DNA was digested with *BamH I *and *Not I*, and blotted to a membrane. The membrane was hybridized with Probe #2. Lane 1, *y*^*1*^*w*^*67c23 *^wild-type control; lane 2, DNA from the donor *P{>w*^*hs*^,Pld*>} flies; lane 3, DNA from the heterozygote line 3 candidate; lanes 4–6, DNA from the homozygote lines 3, 8 and 9 candidates, respectively. The wild-type band in the *yw *control is observed as a doublet because of a polymorphism or insertion in one of the parental chromosomes between *Pld *and *bin3*. Southern blot hybridization using probe #1 confirmed the structure of the mutant allele at the 5' end of the insertion site for all three lines as diagrammed (not shown). None of the *Pld*^*null *^lines retained the I-*SceI *restriction sequence at the site of integration in exon 5: in each case, the wild-type sequence was restored.

The insertional, or ends-in, homologous recombination method [32] was used to mutate the *Pld *gene. For gene targeting (Fig. 5), a 4.8-kb genomic fragment of *Pld *beginning in exon 2 at amino acid 149 of the ORF and terminating 18 amino acids before the stop codon in exon 9 was engineered to carry a recognition site for the I-*SceI *endonuclease and two stop codons within exon 5. Seven independent *Pld *targeting events were recovered after screening 60,000 flies. Of the 7 events, only 3 had the expected genomic locus organization (Fig. 5) and the presence of the two introduced stop codons. The remaining 4 repaired the inserted DNA sufficiently distal to the insertion site that one or both stop codons were converted back to the wild-type sequence. The recombination event generated a tandem duplication of the *Pld *locus resulting in two truncated (C-terminally truncated and N-terminally truncated, respectively) *Pld *genes. Analysis of *Pld *mRNA from *Pld*^*null *^mutant flies by RT-PCR indicated that only the first gene (which contained a functional promoter) was expressed, and trans-splicing did not occur (not shown). The corresponding truncated Pld protein generated would be 1001 amino acids long and contain only 1 HKD domain, and thus would be inactive, since as demonstrated previously [25], two functional HKD domains are necessary for PLD activity. Expression of this truncated protein should not result in phenotypic consequences, since, as described above, overexpression of a presumptively inactive Pld allele (mutated in the second HKD motif (Pld-H1095N)) did not increase PLD activity in tissue culture systems and did not cause embryonic lethality.

*Pld*^*null *^flies could be recovered as homozygotes, but displayed a 56% ± 6% reduction in expected viability in comparison to heterozygous flies as determined by mating heterozygous null flies (*Pld*^*null*^*/CyO *× *Pld*^*null*^*/CyO*; *CyO *is the curly wing balancer). This result indicates that zygotic transcription of *Pld *is important, since the maternal *Pld *mRNA was present. A larger decrease in viability (90% ± 3% reduction) was observed when homozygous null females, collected from the heterozygous stock, were crossed with heterozygous males (*Pld*^*null*^*/Pld*^*null *^× *Pld*^*null*^*/CyO*), indicating that maternal *Pld *is also important. It should be noted, however, that the effects on viability progressively decreased after maintaining the line for several generations as a homozygous *Pld*^*null *^stock (data not shown), suggesting that compensatory mechanisms that offset the loss of *Pld *become activated or selected for.

To determine the embryonic developmental stage at which the *Pld*^*null *^flies were arresting, we monitored the progressive development of living *Pld*^*null *^embryos. Of 53 *y*^*1*^*w*^*67c23 *^control embryos, almost all progressed normally through cellularization and gastrulation (the 3 that did not were either unfertilized or arrested early in mitosis). Of 78 *Pld*^*null *^embryos, almost half (37) exhibited visible morphological defects prior to or during cellularization, and 15 displayed abnormal gastrulation (which could have ensued from abnormalities initiated during cellularization). However, it should be noted that of the 15 that exhibited abnormal gastrulation (as characterized by a thin and shortened germ band), 3 were still able to progress to the larval stage and hatch. Taken together, these results indicate that *Pld *facilitates the normal progression through early embryogenesis.

### Pld deficiency results in Golgi and/or Golgi-derived membrane vesicles increased in size

**Figure 6:**
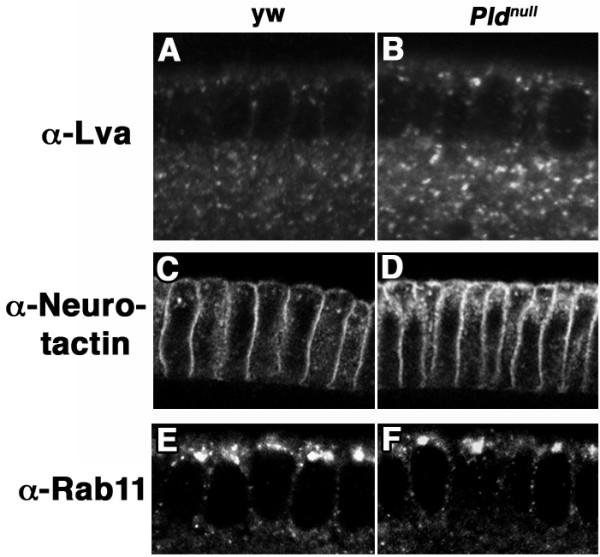
**Lva-marked Golgi vesicles display increased size in Pld^*null *^embryos and plasma membrane insertion of Neurotactin (Nrt) is defective, but Rab11 localization is unaltered**. Cellularizing embryos were immunostained with affinity-purified antisera to visualize trafficking proteins and subcellular compartments. A, B. Anti-Lva antisera to visualize Golgi vesicles A. Two *y*^*1*^*w*^*67c23 *^control embryos, representative of 82 examined, illustrate Lva-stained vesicles present both apically and basally. B. Two *Pld*^*null *^embryos, representative of 75 examined, reveal an increased intensity of Lva staining and increased size of Lva-marked Golgi vesicles. Images were taken at or near the embryonic equator. The differences in Lva-staining intensity were reproducible across multiple experiments and batches of embryos. Aggregate size distributions were quantified on 12 *y*^*1*^*w*^*67c23 *^control and 12 *Pld*^*null *^embryos using NIH Image J software. C, D. Cellularizing embryos were heat-fixed and immunostained with mouse monoclonal anti-Nrt. C. In representative *y*^*1*^*w*^*67c23 *^control embryos (two are shown), Nrt accumulates at the plasma membrane and in small vesicles. D. In representative *Pld*^*null *^embryos, in contrast, neurotactin accumulates subapically and apicolaterally. In total, 36 embryos from multiple experiments were examined with similar results. E, F. Cellularizing embryos were heat-fixed and immunostained with rat polyclonal anti-Rab11.

To examine potential mechanisms responsible for the abnormal progression of *Pld*^*null *^embryos during embryogenesis, we assessed the subcellular localization of Lava Lamp (Lva), a Golgi-associated protein essential for cellularization that facilitates interaction between Golgi secretory vesicles and the cytoskeleton [6]. In wild-type cellularizing embryos, Lva localized to Golgi vesicles, particularly in basal regions of the forming cells (Fig. 6A, B). In contrast, *Pld*^*null *^embryos exhibited increased fluorescence intensity (1.7+0.2-fold per similar vesicle area) upon staining with anti-Lva antibody, and the Lva-stained Golgi vesicles were increased in size (8.3 ± 1.9-fold). This observation of abnormal Golgi structure suggests a role for Pld in facilitating the Golgi fissioning required to generate the vesicles destined to be transported via Lva to the embryonic cortex.

### Pld deficiency results in accumulation of Neurotactin-containing vesicles at or near the plasma membrane

The transmembrane protein Neurotactin (Nrt), which is synthesized de novo during cellularization, traffics through the secretory pathway and translocates to the apical plasma membrane via Golgi-derived vesicles, following which it undergoes endocytosis and re-fusion into lateral membranes via the recycling endosomal pathway. Vesicles bound for lateral membranes may also travel there via the Rab11 endosomal pathway without first trafficking to the plasma membrane [8]. In Pld-deficient embryos, Nrt-containing vesicles were observed to accumulate throughout the apical cytoplasm and in a large subapical/apicolateral compartment, in large part juxtaposed to the plasma membrane (compare Fig. 6D with 6C). A similar appearing pattern of mis-localization is observed in *shibire *mutant embryos [8]; in this setting, the aggregating vesicles co-localize with Rab11, suggesting that they are composed of recycling endosomes unable to fuse into lateral membranes. *shibire *is a dynamin isoform required for budding from recycling endosomes. However, the aggregating vesicles in Pld-deficient embryos did not co-localize with Rab11 (data not shown), nor were the Rab11-containing vesicles observed to be altered significantly in localization or morphology (Fig. 6E, F). Moreover, the Nrt vesicle aggregates did not co-localize with a marker for Golgi (data not shown). Thus, Nrt appears to exit the Golgi into secretory vesicles and translocate apically, but then is not inserted into the plasma membrane, thereby suggesting that, in addition to its role in Golgi vesicle fission, Pld mediates vesicle fusion at the plasma membrane during cellularization. As another possibility, Pld may mediate fusion of post-Rab11 vesicles into the lateral membrane.

### Cellularization is delayed in Pld^null ^embryos

Since the *Pld*^*null *^mutants displayed cellularization defects consisting of altered Golgi structure and post-Golgi trafficking, we examined whether these defects were detrimental for cellularization. Single embryos from an outcrossed *Pld*^*null *^stock were analyzed using video microscopy starting at pole cell formation through the end of cellularization. Three of the six *Pld*^*null *^embryos exhibited a substantial delay during cellularization (Table 1), taking an average of 28% longer to complete the process (87 minutes versus 68 minutes), and a fourth was significantly delayed. Two of the six *Pld*^*null *^embryos were wild-type in their progression through cellularization, suggesting incomplete penetrance of the phenotype. Nonetheless, the delay in cellularization in the majority of Pld-deficient embryos indicates that the presence of Pld facilitates the progression of cellularization in a timely manner.

**Table 1:** Cellularization is delayed in homozygous *Pld*^*null *^embryos

**Embryo**	**Total time from cellularization start to pole cell migration (min:sec)**	
*yw *#1	65:49	
*yw *#2	68:09	
*yw *#3	71:15	

**Embryo**	**Total time from cellularization start to pole cell migration (min:sec)**	**increase in time required for cellularization**

*Pld*^*null *^#1	69:42	1.9%
*Pld*^*null*^#2	86:26	26.4%
*Pld*^*null*^#3	69:30	1.6%
*Pld*^*null*^#4	88:33	29.4%
*Pld*^*null*^#5	75:12	9.9%
*Pld*^*null*^#6	86:21	26.2%

The embryonic development of single embryos from the time of pole cell formation to the start of gastrulation was recorded using a stereomicroscope with an attached video recorder. The videos were analyzed from the start of cellularization (marked by initial membrane invagination and pole cell formation) to the beginning of pole cell migration and gastrulation. *Pld*^*null *^embryos were collected from cages set up with homozygous *Pld*^*null *^males and females from an outcrossed heterozygous *Pld*^*null *^stock.

## Discussion and Conclusions

The role of lipid signaling enzymes in the regulation of membrane trafficking during cellularization is largely unknown. We report here that Phospholipase D plays an important role in *Drosophila *embryonic cellularization, using the experimental approaches of overexpression and gene targeting. The severity of the gene targeting phenotype is potentially understated since *Pld*^*null *^flies appear to activate compensatory mechanisms: heterozygous null flies, when first crossed, generate few homozygous offspring (10% of the expected frequency), but continued breeding of the surviving null flies yields a stock with wild-type levels of viability. Such compensation has been observed for lipid signaling [e.g. [33,34]] and in general in other *Drosophila *systems [35,36]). Supporting the possibility of compensatory mechanisms, preliminary experiments using PLD RNAi on wild-type embryos, a setting in which compensatory mechanisms would not easily be activated, elicited in a nearly complete (82%) inhibition of cellularization (data not shown). Although there is only one PLD isoform in *Drosophila*, which rules out redundant coverage via a closely-related family member, PA levels could be supported through at least three other mechanisms – increasing activity of DAG Kinase (which generates PA through phosphorylation of DAG), decreasing activity of phosphatidic phosphohydrolases (which mediate the reverse reaction), or increasing activity of LysoPhosphatidic Acetyl Transferases (which convert LysoPhosphatidic acid to PA). A question for the future will be to determine if any of the multiple members of these large gene families are altered in expression in Pld-deficient flies.

Only recently has the importance of targeted membrane secretion in cellularization been recognized. Lecuit and Wieschaus (2000) showed that membrane growth during cellularization occurs in large part through the regulated mobilization of ER- and/or Golgi-derived membrane populations that insert into precise locations along the plasma membrane. We report here that on a gross level, loss of Pld impairs progression through cellularization. Presumably underlying this observation, two consequences of Pld deficiency on vesicle trafficking were observed: altered morphology of the Golgi apparatus, from which secretory vesicles are derived, and diminished insertion of the secretory vesicles into the apical and apicolateral plasma membrane.

PLD has been reported to play roles in vesicle trafficking in other organisms. In yeast, PLD is required to form the prospore membrane during meiosis via fusion of secretory pathway-derived vesicles [17] and for the generation of secretory vesicles in mutant strains lacking Sec14p [33]. In mammalian cells, PLD1 facilitates secretory vesicle generation [14] and fusion into the plasma membrane [18], whereas PLD2 has been proposed to facilitate endocytosis [37]. The results we describe here would be consistent with the roles reported for PLD1 and for yeast PLD, and establish the first animal model that can be used to explore the function of PLD *in vivo*. We have described one other use of this mode, in the exploration of the role of PLD in phototransduction [24].

Of note, Pld does not exhibit steady-state localization in the Golgi. However, studies on mammalian PLD have suggested that both PLD1 and PLD2 cycle through a succession of subcellular membrane locations including the plasma membrane, recycling and outward bound endosomes, and the Golgi, and that they undertake functional roles at multiple steps during the transit [16,38]. The steady-state pattern of localization reflects just the slowest step in the cycle, which differs in different cell types or as a consequence of the initiation of signaling events. Our observation that Pld exhibits both vesicular and plasma membrane localization in mammalian cells (Fig. 4) suggests that it too is likely to function at multiple subcellular sites under appropriate circumstances.

Future questions include how Pld is activated during cellularization and how alterations in Pld activity change the morphological structure of the Golgi. Numerous questions remain as to how fusion-competent vesicles are generated from the Golgi and undergo translocation to sites of incorporation into the extending membranes. Further exploration of the role of Pld in this process should lead to new insights.

## Methods

### General reagents

Phospholipids were purchased or prepared as described [27]. All other reagents were obtained from standard sources.

### Plasmid construction

The *pCGN:Pld *and *pCGN:Pld-H1095N *mammalian expression plasmids were constructed using the coding region of cDNA GH07346. From the above plasmids, the resulting 3.9-kb *Pld *coding region was excised using *Xba I *and *BamH I *and ligated into the *EcoR I/Bgl II *sites of *pUAS-T *(Brand and Perrimon, 1993) to generate *pUAS:Pld and pUAS-Pld-HN*, respectively. Prior to ligation, the *Xba I *and *EcoR I *sites were filled in.

*Homologous recombination targeting construct P{>w*^*hs*^,Pld*>}. See Fig. 5 for schematic. A *BamH I *fragment of BAC clone RCP198.08AII that contained *Pld *was inserted into pCaSpeR4. A 4.8-kb *BamH I*-*Sac I *fragment containing *Pld *exons 2–9 was cloned into *pBS(Not) *(gift of K. Golic, U. Utah). Two mutated *pBS(Not): Pld *plasmids, each containing a different stop codon in exon 5 (at Pld amino acids 616 and 1002, respectively) were generated using QuickChange. *Pld *fragments were generated from both plasmids by PCR. Each fragment encoded a different stop codon, terminated at the exon 5 end in a phosphorylated half I-*SceI *recognition site (ttatcccta or cagggtaat, respectively), and contained either *Not I *or *Acc65 I *at the opposite end. The fragments were digested with either *Not I *or *Acc65 I *and inserted as a 3-way ligation into a similarly restricted pTV2 vector (gift of K. Golic). The blunt ligation of the half-I-*Sce I *sites generated the full 18-bp site.

### Fly strains and crosses

*Drosophila melanogaster *strains were maintained on standard cornmeal/yeast/sugar and agar media. Transgenic lines carrying *P{UAS-Pld}*, *P{UAS-Pld-H1095N}*, or *P{>w*^*hs*^,Pld*>} were generated by embryo transformation of *y*^*1*^*w*^*67c23 *^flies [39]. Flies carrying the *P{GAL4-nos.NGT}11 *[31] and the *P{GAL4-nos.NGT}40; P{GAL4-nos.NGT}A *[40] drivers were used for viability assays as described by Wheeler *et al*. (2002). Three different *P{UAS-Pld} *and *P{UAS-Pld-H1095N} *insertion lines were used. All counts were performed in duplicate at 25°C. For homologous recombination experiments, targeting was performed using a single *P{>w*^*hs*^,Pld*>} donor on the third chromosome. *P{>w*^*hs*^,Pld*>} virgin females were crossed to *w (v);; P [ry*^+^*, 70FLP] P [v*^+^*, 70I-SceI]*/TM3, Ser males (gift of K. Golic), generating *P [ry*^+^*, 70FLP] P [v*^+^*, 70I-SceI]/P{>w*^*hs*^,Pld*>} progeny. Eggs were collected for 24 hours and heat shocked between 24 and 48 hours for 1 hour at 38°C. After hatching, the flies carrying one copy of P{>*w*^*hs*^,Pld*>} and *70FLP *and *70I-SceI *were crossed to a *70FLP *line that has strong constitutive expression of FLP (gift of K. Golic). Flies were screened for *w*^+ ^non-mosaic eyes, followed by translocation of the *w*^*hs *^from the third to the second chromosome by crossing to Balancer stocks. Outcrosses against wild-type strains were performed prior to functional analyses.

### Molecular analysis of the Pld^null^

Genomic DNA was extracted using standard protocols. Southern blot hybridization was performed using the probes shown in Fig. 5. Allele specific PCR and sequencing were performed to assay for the presence of the introduced stop codon mutations.

### Quantitative PCR

Total RNA was extracted from *Canton-S *embryos at different embryonic stages using the StrataPrep Total RNA Miniprep Kit (Stratagene). cDNA was synthesized using the First Strand cDNA Synthesis Kit for RT-PCR (AMV) (Roche). Quantitative PCR measurements were performed using a LightCycler and the LightCycler Faststart DNA Master SYBR Green I kit (Roche). Primers used for the amplification of *Pld *cDNA were 5'-aggagacggacgatgatgag (exon 2) and 5'-cgattgtgtacagattggg (spans the exon 2-exon 3 boundary). Primers 5'-tacaggcccaagatcgtgaa and 5'-tctccttgcgcttcttgga were used for the amplification of *rp49*. Samples were run in triplicate and standardized using *Pld *and *rp49 *plasmid DNA.

### PLD assays

PLD cDNAs were transiently overexpressed in COS7 or HEK-293 cells using the mammalian expression vector *pCGN*. In vivo assays were performed as described previously [41].

### Immunological techniques and confocal microscopy

A 405-bp *Pld *cDNA fragment corresponding to the amino-terminal 135 amino acids of the Pld protein was cloned into the *pET32a *bacterial expression vector (Novagen). The resulting His-Trx-Pld fusion protein was purified on Ni^+^-resin and used to immunize rabbits (Covance). The antisera was antigen affinity-purified. An anti-Pld peptide antisera directed against the C-terminus was also generated in rabbits and affinity purified. The affinity-purified antibodies were used at 1 μg/ml. Other primary antibodies used included mouse monoclonal anti-actin (1:200; ICN Biomedical, Inc.), mouse monoclonal anti-p120 (1:250; CalBiochem), mouse monoclonal anti-neurotactin (10 μg/ml; BP106 clone, Hybridoma Bank), rabbit polyclonal anti-Lava Lamp (1:5000; gift of W. Sullivan), and rat polyclonal anti-rab11 (1:2000; gift of R. Cohen). Fluorescent secondary antibodies were from Molecular Probes. Propidium iodide was used at 1 μg/ml. Western blot analysis was performed according to standard protocols. Whole mount antibody staining on embryos was performed as described [42]. Both anti-Pld and anti-neurotactin required heat-fixation of embryos. Images were captured using a Leica TCS SP2 confocal microscope and processed using Adobe Photoshop.

### Transfection and immunofluorescent staining of COS7 cells

COS7 cells were maintained, transfected, and immunostained using standard protocols. Primary antibodies included: rat anti-HA (1:300), rabbit anti-HA (1:100), rabbit anti-calreticulin (1:200, StressGen), and mouse anti-transferrin receptor (1:150). Fluorescent secondary antibodies were from Molecular Probes.

### Embryonic staging for the Pldnull mutant

*y*^*1*^*w*^*67c23 *^and *Pld*^*null *^mutant flies were allowed to lay eggs on apple juice plates for 1 hour. The plates were then aged for 1.5 hours, following which the eggs were transferred to new plates and covered with halocarbon oil 57. The embryos were staged, followed through the initial stages of gastrulation using a dissecting microscope, placed at 25°C for 24 hours, and analyzed for the presence of larva.

### Video microscopy on homozygous Pld^null ^embryos from the outcrossed Pld^null ^stock

The *Pld*^*null *^stock was outcrossed for five generations and then balanced over CyO. Homozygous *Pld*^*null *^embryos were collected from a cage set up with homozygous (straight-winged) males and females collected from the heterozygous *Pld*^*null *^stock. Embryos prior to pole cell formation were transferred to a drop of halocarbon oil57 on a Petriperm dish and cover sliped. Embryonic development from the time of pole cell formation to the start of gastrulation was recorded using a stereomicroscope with an attached video recorder.

## Authors' contributions

ML generated the Pld antisera, performed the viability, videomicroscopy, western blot analysis, Pld immunolocalization and marker analyses, generated the transgenic rescue strain, and co-drafted the manuscript. HJ generated the Pld^*null *^strain using homologous recombination, performed viability and videomicroscopy analyses, and co-drafted the manuscript. SJ performed the RNAi experiments and analysis. JC cloned the PLD gene. OR performed 5' RACE to obtain full length cDNA clones, performed transcriptional initation analysis, and examined IRES activity of the 5' untranslated region. VO performed the RT-PCR analysis of emboryonic expression. YMA and GD analyzed the biochemical activity of Pld in mammalian cells. JPG and MAF made substantial contributions to the conception and design of the project, the interpretation of the data, and the subsequent revision of the manuscript for publication. All of the authors reviewed the final version of the manuscript and gave approval for it.

## References

[B1] Fullilove SL, Jacobson AG (1971). Nuclear elongation and cytokinesis in Drosophila montana. Dev Biol.

[B2] Turner FR, Mahowald AP (1977). Scanning electron microscopy of Drosophila melanogaster embryogenesis. II. Gastrulation and segmentation. Dev Biol.

[B3] Loncar D, Singer SJ (1995). Cell membrane formation during the cellularization of the syncytial blastoderm of Drosophila. PNAS.

[B4] Burgess RW, Deitcher DL, Schwarz TL (1997). The synaptic protein syntaxin 1 is required for cellularization of Drosophila embryos. J Cell Biol.

[B5] Lecuit T, Wieschaus E (2000). Polarized Insertion of New Membrane from a Cytoplasmic Reservoir during Cleavage of the Drosophila Embryo. Journal of Cell Biology.

[B6] Sisson JC, Field C, Ventura R, Royou A, Sullivan W (2000). Lava lamp, a novel peripheral golgi protein, is required for Drosophila melanogaster cellularization. J Cell Biol.

[B7] Lecuit T, Samanta R, Wieschaus E (2002). slam encodes a developmental regulator of polarized membrane growth during cleavage of the Drosophila embryo. Dev Cell.

[B8] Pelissier A, Chauvin JP, Lecuit T (2003). Trafficking through Rab11 endosomes is required for cellularization during Drosophila embryogenesis. Curr Biol.

[B9] Crawford JM, Harden N, Leung T, Lim L, Kiehart DP (1998). Cellularization in Drosophila melanogaster is disrupted by the inhibition of rho activity and the activation of Cdc42 function. Dev Biol.

[B10] Padash Barmchi M, Rogers S, Hacker U (2005). DRhoGEF2 regulates actin organization and contractility in the Drosophila blastoderm embryo. J Cell Biol.

[B11] Riggs B, Rothwell W, Mische S, Hickson GR, Matheson J, Hays TS, Gould GW, Sullivan W (2003). Actin cytoskeleton remodeling during early Drosophila furrow formation requires recycling endosomal components Nuclear-fallout and Rab11. J Cell Biol.

[B12] McDermott M, Wakelam MJ, Morris AJ (2004). Phospholipase D. Biochem Cell Biol.

[B13] Ktistakis NT, Brown HA, Waters MG, Sternweis PC, Roth MG (1996). Evidence that phospholipase D mediates ADP ribosylation factor- dependent formation of Golgi coated vesicles. J Cell Biol.

[B14] Chen YG, Siddhanta A, Austin CD, Hammond SM, Sung TC, Frohman MA, Morris AJ, Shields D (1997). Phospholipase D stimulates release of nascent secretory vesicles from the trans-Golgi network. J Cell Biol.

[B15] Choi WS, Kim YM, Combs C, Frohman MA, Beaven MA (2002). Phospholipases D1 and D2 regulate different phases of exocytosis in mast cells. J Immunol.

[B16] Du G, Altshuller YM, Vitale N, Huang P, Chasserot-Golaz S, Morris AJ, Bader MF, Frohman MA (2003). Regulation of phospholipase D1 subcellular cycling through coordination of multiple membrane association motifs. J Cell Biol.

[B17] Rudge SA, Morris AJ, Engebrecht J (1998). Relocalization of Phospholipase D activity mediates membrane formation during meiosis. Journal of Cell Biology.

[B18] Vitale N, Caumont AS, Chasserot-Golaz S, Du G, Wu S, Sciorra VA, Morris AJ, Frohman MA, Bader MF (2001). Phospholipase D1: a key factor for the exocytotic machinery in neuroendocrine cells. Embo J.

[B19] Huang P, Altshuller YM, Chunqiu Hou J, Pessin JE, Frohman MA (2005). Insulin-stimulated Plasma Membrane Fusion of Glut4 Glucose Transporter-containing Vesicles Is Regulated by Phospholipase D1. Mol Biol Cell.

[B20] Honda A, Nogami M, Yokozeki T, Yamazaki M, Nakamura H, Watanabe H, Kawamoto K, Nakayama K, Morris AJ, Frohman MA, Kanaho Y (1999). Phosphatidylinositol 4-phosphate 5-kinase alpha is a downstream effector of the small G protein ARF6 in membrane ruffle formation. Cell.

[B21] Rose K, Rudge SA, Frohman MA, Morris AJ, Engebrecht J (1995). Phospholipase D signaling is essential for meiosis. Proc Natl Acad Sci U S A.

[B22] Hammond SM, Altshuller YM, Sung TC, Rudge SA, Rose K, Engebrecht J, Morris AJ, Frohman MA (1995). Human ADP-ribosylation factor-activated phosphatidylcholine-specific phospholipase D defines a new and highly conserved gene family. J Biol Chem.

[B23] Colley WC, Sung TC, Roll R, Jenco J, Hammond SM, Altshuller Y, Bar-Sagi D, Morris AJ, Frohman MA (1997). Phospholipase D2, a distinct phospholipase D isoform with novel regulatory properties that provokes cytoskeletal reorganization. Curr Biol.

[B24] LaLonde MM, Janssen H, Rosenbaum E, Choi SY, Gergen JP, Colley NJ, Stark WS, Frohman MA (2005). Regulation of phototransduction responsiveness and retinal degeneration by a phospholipase D-generated signaling lipid. J Cell Biol.

[B25] Sung TC, Roper RL, Zhang Y, Rudge SA, Temel R, Hammond SM, Morris AJ, Moss B, Engebrecht J, Frohman MA (1997). Mutagenesis of phospholipase D defines a superfamily including a trans- Golgi viral protein required for poxvirus pathogenicity. EMBO J.

[B26] Miller RR, Yates JW, Geer BW (1993). Dietary ethanol stimulates the activity of phosphatidylcholine-specific phospholipase D and the formation of phosphatidylethanol in Drosophila melanogaster larvae. Insect Biochem Mol Biol.

[B27] Du G, Altshuller YM, Kim Y, Han JM, Ryu SH, Morris AJ, Frohman MA (2000). Dual requirement for rho and protein kinase C in direct activation of phospholipase D1 through G protein-coupled receptor signaling. Mol Biol Cell.

[B28] Mazumdar A, Mazumdar M (2002). How one becomes many: blastoderm cellularization in Drosophila melanogaster. Bioessays.

[B29] Chen YG, Shields D (1996). ADP-ribosylation factor-1 stimulates formation of nascent secretory vesicles from the transgolgi network of endocrine cells. J Biol Chem.

[B30] Bi K, Roth MG, Ktistakis NT (1997). Phosphatidic acid formation by Phospholipase D is required for transport from the endoplasmic reticulum to the Golgi complex. Current Biology.

[B31] Tracey WD, Ning X, Klingler M, Kramer SG, Gergen JP (2000). Quantitative analysis of gene function in the Drosophila embryo. Genetics.

[B32] Rong YS, Golic KG (2000). Gene targeting by homologous recombination in Drosophila. Science.

[B33] Xie Z, Fang M, Faulkner AJ, Sternweis PC, Engebrecht J, Bankaitis V (1998). Phospholipase D activity is required for suppression of yeast phosphatidylinositol transfer protein defects. PNAS.

[B34] Padron D, Wang YJ, Yamamoto M, Yin H, Roth MG (2003). Phosphatidylinositol phosphate 5-kinase Ibeta recruits AP-2 to the plasma membrane and regulates rates of constitutive endocytosis. J Cell Biol.

[B35] Henry JR, Harrison JF (2004). Plastic and evolved responses of larval tracheae and mass to varying atmospheric oxygen content in Drosophila melanogaster. J Exp Biol.

[B36] Reis T, Edgar BA (2004). Negative regulation of dE2F1 by cyclin-dependent kinases controls cell cycle timing. Cell.

[B37] Du G, Huang P, Liang BT, Frohman MA (2004). Phospholipase D2 localizes to the plasma membrane and regulates angiotensin II receptor endocytosis. Mol Biol Cell.

[B38] Sciorra VA, Rudge SA, Wang J, McLaughlin S, Engebrecht J, Morris AJ (2002). Dual role for phosphoinositides in regulation of yeast and mammalian phospholipase D enzymes. J Cell Biol.

[B39] Rubin GM, Spradling AC (1982). Genetic transformation of Drosophila with transposable element vectors. Science.

[B40] Wheeler JC, VanderZwan C, Xu X, Swantek D, Tracey WD, Gergen JP (2002). Distinct in vivo requirements for establishment versus maintenance of transcriptional repression. Nat Genet.

[B41] Du G, Morris AJ, Sciorra VA, Frohman MA (2002). G-protein-coupled receptor regulation of phospholipase D. Methods Enzymol.

[B42] Kosman D, Small S, Reinitz J (1998). Rapid preparation of a panel of polyclonal antibodies to Drosophila segmentation proteins. Dev Genes Evol.

